# Optical Technologies for Healthcare and Wellness Applications

**DOI:** 10.1155/2019/1321348

**Published:** 2019-03-17

**Authors:** Ting Li, Yu Shang, Weiqing Ge

**Affiliations:** ^1^Institute of Biomedical Engineering, Chinese Academy of Medical Sciences and Peking Union Medical College, Tianjin 300192, China; ^2^North University of China, Key Laboratory of Instrumentation Science and Dynamic Measurement, Taiyuan, China; ^3^Department of Physical Therapy, Youngstown State University, Youbgstown, Ohio 44555, USA

The motivation for research and development in healthcare and wellness is necessary for successfully addressing healthcare challenges, reducing healthcare costs while maintaining high quality patient services, and shifting healthcare priorities from treatment to prevention [1]. Technologies play a more and more important role in healthcare and wellness. For this special issue, the editorial team focused on the application of optoelectronic technologies in healthcare and wellness, and the most representative 9 research papers were identified from the manuscripts received.

With the advancements in the wireless communication technologies of data communication media and biosensors to measure bioengineering signals of a human body, the wearable mobile computing technology is developed and utilized in various areas from healthcare for senior citizens to sports activity [2]. Portable and wearable technologies allow monitoring to be extended to the community. They have been used in many research and clinical applications, including monitoring health, monitoring elderly and frail individuals and individuals with neurological disorders, measuring levels of physical activity in disease-related studies, and developing behavioral interventions. Combined, portable, and wearable optoelectronic technology creates unique continuous behavior and physiological monitoring in the home and community.

The goal of this special issue is to highlight the latest applications in portable and wearable optoelectronics, with a focus on the technology's health and healthcare applications. The included issues cover the latest developments in the healthcare and health framework for small size and wearable medical optoelectronics, biomedical sensor technology, clinical applications, measurement algorithms, signal processing, statistics, problems and solutions, as well as opportunities and challenges.

In this special issue, an innovative wearable device, utilizing optoelectronic technology, is developed to help people meditate to relieve mental fatigue. The researchers also applied biomedical sensor technology to design an anti-crosstalk sensor that plays an important role in the development of pulse oximetry. Besides, some important algorithms and modeling are applied to optical technologies for healthcare and wellness applications. To be specific, researchers applied piecewise linear algorithm, regression tree algorithm, and Gaussian modeling features to the force analysis of joint-assisted pelvic support walking robot and accurate measurement of blood pressure and maternal physiological changes during pregnancy. New ultrasound-specific guidewire and Neuroinformation Database for Developing Networks (NDDN) play an important role in studying physiological and mental health. Moreover, Raman scattering has been reviewed as being used in the current state of cancer screening and unlimited potential in the future.
